# Supplementary material for: Compact all-fiber quantum-inspired LiDAR with over 100 dB noise rejection and single photon sensitivity
Source: Nat Commun. 2023 Sep 2;14:5344. doi: 10.1038/s41467-023-40914-6 (PMC10475127; doi:10.1038/s41467-023-40914-6)
Supplement: Supplementary file 1 — Supplementary Information [file 41467_2023_40914_MOESM1_ESM.pdf]

Compact All-Fiber Quantum-Inspired LiDAR with over 100dB Noise Rejection and Single Photon Sensitivity: Supplementary Material

August 14, 2023

1 Supplementary Note 1 - Comparison of Different Correlation-based LiDAR Protocols

In this section, we give a brief review of different types of classical and quantum LiDARs that are based on correlation or similar concepts. Specifically, we compare their performance metrics in terms of noise resilience, probe light characteristics, receiver design, and ranging capability, as summarized in the following table and figure:

Table S1: Comparison between different LiDAR technologies

LiDAR Mechanism	chaotic-QFC	QTC[1, 2, 3]	p-QFC[4]
Noise Rejection	107dB (0.1s integration)	43dB	40dB
Probe power	Arbitrary	Single Photon	Arbitary
Detector	Frequency Conversion	Photon Counting	Frequency Conversion
Detector Saturable	No	Yes	No
Probe Characteristics	Chaotic	Chaotic	Pulsed
Resolution/Range	um/Optical Scanning	cm/Long Range	mm/Optical Scanning

LiDAR Mechanism	FMCW[5]	RF Correlation[6, 7, 8]	ToF
Noise Rejection	$\simeq$20dB	20dB (25ns integration)	0dB
Probe power	Arbitary	Arbitary	Arbitary
Detector	Optical Interference	Direct Detection	Direct Detection
Detector Saturable	Yes	Yes	Yes
Probe Characteristics	Ramping Frequency	Chaotic	Pulsed
Resolution/Range	cm/Long Range	cm/Long Range	cm/Long Range

Table S1: The noise rejection is quantified as the ratio between noise and probe power when the peak to side lobe level (PSL) is 3dB (for a specified integration time, if applicable). A LiDAR receiver is considered not saturable if noise light can be optically separated from the probe light before photo-detection. A LiDAR is considered long-range if the target distance is determined through post data processing, otherwise, it requires optical scanning to determine the target distance.

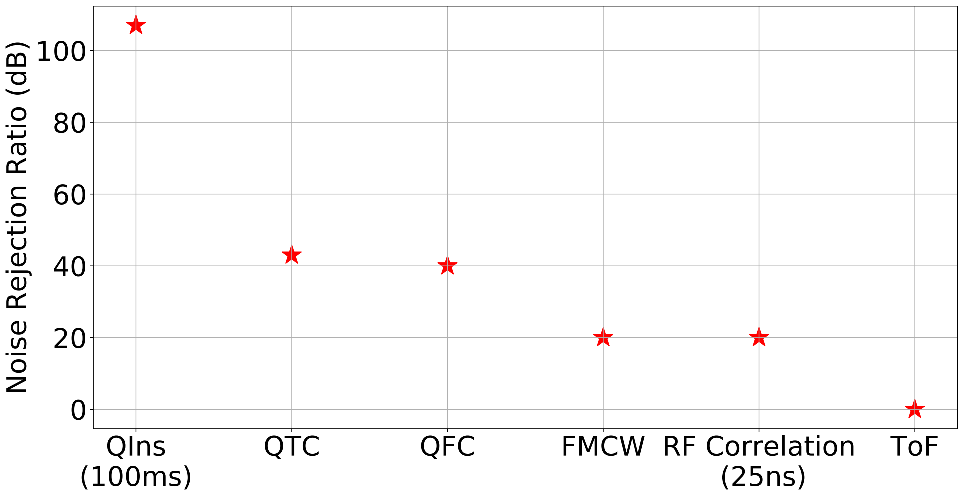

Fig. S1: Noise rejection comparison between different LiDAR technologies.

Quantum Temporal/Spectral Correlation (QTC)

The QTC protocol [1, 2, 3] relies on phase insensitive measurement (photon counting) of time-frequency correlation between entangled probe and reference photons. In QTC, the entanglement between the probe and reference photon is not fully analyzed therefore the achievable noise rejection is lower than QI. However, the phase-insensitive nature of QTC allows for ranging through post-processing of the photon counting data. Detector saturation and ranging resolution of QTC are limited by the single photon detector dead-time and timing resolution. The entangled light source used in QTC is chaotic and will not interfere with other identical sources.

Pulsed Quantum Frequency Conversion (p-QFC) LiDAR

The p-QFC LiDAR [4] uses the quantum frequency conversion (QFC) technique to selectively convert pulsed probe light into a different frequency band. Therefore, only temporal but not spectral discrimination of noise is provided. The target distance is determined by scanning the QFC pump pulse delay relative to the probe pulse and millimeter-level resolution can be achieved. Multiple p-QFC LiDARs at work together will induce mutual interference because of identical probe pulses used.

Frequency Modulated Continuous Wave (FMCW) LiDAR

The FMCW LiDAR uses continuous wave probe light whose frequency is periodically ramping. The back-reflected probe light interferes with the local oscillator (LO, time-delayed version of the probe light) and the beating signal reflects both the target's distance and velocity information. Although noise light does not interfere with the LO, it still illuminates the photo-detector and limits the dynamical range and noise rejection. Multiple FMCW LiDARs at work together will induce mutual interference (false alarm and ghost image) [5] because of indistinguishable FMCW sources.

rf Chaotic LiDAR

The rf chaotic LiDAR uses GHz-band-wide chaotic probe light that is generated from nonlinear laser dynamics [6] or random waveform modulation [7, 8]. The measured back-reflected probe light waveform is electronically correlated with the locally stored reference waveform to determine the target distance. The ranging resolution and noise rejection are determined by the probe light bandwidth as well as the detection bandwidth. Noise rejection is done through temporal domain correlation analysis without optically rejecting noise light. Independent rf chaotic LiDAR sources will not interfere with each other provided that their total power does not jam the channel.

Time of Flight (ToF) Pulsed LiDAR

The ToF LiDAR is used as a baseline to compare the characteristics of different LiDAR protocols. In a ToF LiDAR, inband noise cannot be temporally rejected if the target position is not known *a priori.* The ranging resolution is determined by the probe light and detection bandwidth. Multiple ToF LiDARs at work together will induce mutual interference because of identical probe pulses used.

Quantum Illumination (QI)

Quantum illumination requires joint measurement, i.e. coherent interaction of probe and reference light whose phase needs to be stabilized down to a sub-wavelength level. Its performance advantage diminishes as the probe power increase beyond the single photon level. QI is not capable of ranging because it requires the target position to be known *a priori* with sub-wavelength accuracy. Experimental demonstration of QI shows 20% enhancement of SNR with background noise 75dB stronger than the probe light [9].

2 Supplementary Note 2 - Theoretical Modeling of the Sum Frequency Generation Process

In this section, we derive the expressions of the small signal sum frequency generation (SFG) amplitude and spectrum (Eq.(5) (7)) and the c-SFG efficiency in the limit of narrowband phase matching (Eq. (10)).

2.1 Coupled Mode Equations

The SFG process inside a $\chi^{(2)}$medium that has negligible group velocity dispersion is governed by the following equations:

$$(\frac{\partial}{\partial z} + \beta_{SFG}\frac{\partial}{\partial t})A_{SFG}(z,t) = i\gamma A_r(t-\beta z)A_m(z,t) \tag{1}$$

$$(\frac{\partial}{\partial z} + \beta\frac{\partial}{\partial t})A_m(z,t) = i\gamma A_r^*(t-\beta z)A_{SFG}(z,t) \tag{2}$$

where the subscripts m, r, SFG stand for the mixed mode (noise and probe light), reference mode, and SFG mode, respectively. The mixed mode and reference mode are assumed to have the same inverse group velocity β while the SFG light has inverse group velocity β_{SFG}. The amplitude of three interacting modes A_m, A_r, A_{SFG} have the unit of square root photon flux, and γ is the normalized nonlinearity. Since the reference light is used as the pump for the SFG process, its amplitude is assumed to be undepleted throughout the interaction. We shall solve the above equations in three different regimes: small signal SFG, narrowband SFG, and general SFG, as outlined below.

2.2 Small Signal SFG Analysis

In this subsection, we derive the expression of the small signal SFG amplitude in the temporal domain (Eq. (5)). When the reference light flux is relatively low, the depletion of mixed mode light is negligible ($A_m(z,t) = A_m(t-\beta z)$). Then:

$$(\frac{\partial}{\partial z} + \beta_{SFG}\frac{\partial}{\partial t})A_{SFG}(z,t) = i\gamma A_r(t-\beta z)A_m(t-\beta z) \tag{3}$$

To solve this equation, defined moving reference frame (z', T) as:

$$z' = z \qquad T = t - \beta_{SFG}z \qquad A'_{SFG}(z',T) = A_{SFG}(z,t) \qquad \Delta\beta = \beta_{SFG} - \beta \tag{4}$$

Then

$$(\frac{\partial}{\partial z} + \beta_{SFG}\frac{\partial}{\partial t})A'_{SFG}(z', T) = i\gamma A_m(T + z'\Delta\beta)A_r(T + z'\Delta\beta) \tag{5}$$

$$\frac{\partial}{\partial z'}A'_{SFG}(z', T) = i\gamma A_m(T + z'\Delta\beta)A_r(T + z'\Delta\beta) \tag{6}$$

$$A'_{SFG}(L, T) = i\frac{\gamma}{\Delta\beta}(A_m(T)A_r(T)) * \Pi(\frac{T}{\Delta\beta L} + 1/2) \tag{7}$$

where $*$ stands for convolution and $\Pi(t)$ is the unit gate function who is unity for $-1/2 < t \leq 1/2$ and zero otherwise. Since the origin of time and the overall phase of the SFG light is insignificant, we can rewrite the SFG temporal amplitude $A_{SFG}(L, t)$ as:

$$A_{SFG}(L, t) = \frac{\gamma}{\Delta\beta}(A_m(t)A_r(t)) * \Pi(\frac{t}{\Delta\beta L}) \tag{8}$$

which is the desired expression in Eq. (5).

2.3 Random Process Model of Probe, Noise and Reference Light

In this subsection, we introduce the stationary Gaussian random process model for the chaotic probe, reference, and noise light and use it to calculate the small signal SFG spectrum. Probe, noise, and reference light are modeled as stationary Gaussian random processes (assuming no relative delay and dispersion of probe and reference light).

$$A_p(t) = \sqrt{\frac{P_p}{2}}(x(t) + iy(t)) \qquad A_r(t) = \sqrt{\frac{P_r}{2}}(x(t) - iy(t)) \qquad A_n(t) = \sqrt{\frac{P_n}{2}}(x_n(t) + iy_n(t)) \tag{9}$$

where P_p, P_r, P_n specify the probe, reference, and noise photon flux, respectively. The independent Gaussian random processes $x(t), y(t), x_n(t), y_n(t)$ are characterized by their covariance functions:

$$\langle x(t)x(t')\rangle = \langle y(t)y(t')\rangle = \exp(-\frac{\sigma^2(t-t')^2}{2}) \tag{10}$$

$$\langle x_n(t)x_n(t')\rangle = \langle y_n(t)y_n(t')\rangle = \exp(-\frac{\sigma^2(t-t')^2}{2}) \tag{11}$$

where σ is the bandwidth of the probe, reference, and noise light. Here we choose a Gaussian-shaped correlation function for algebraic simplicity but a correlation function corresponding to an arbitrary power spectral density can also be used. Since the SFG amplitude is also a stationary random process, its power spectral density is given by its correlation function's Fourier transform:

$$S(\omega) = \frac{1}{2\pi}\mathcal{F}\{\langle A_{SFG}(t)A^*_{SFG}(t')\rangle\} \tag{12}$$

$$= \frac{1}{8\pi}\gamma^2 L^2 \text{sinc}(\frac{\Delta\beta L\omega}{2})\mathcal{F}\{\langle P_r P_p(x^2(t) + y^2(t))(x^2(t') + y^2(t'))$$
$$+ P_r P_n(x_n(t) + iy_n(t))(x_n(t') - iy_n(t'))(x(t) + iy(t))(x(t') - iy(t'))\rangle\} \tag{13}$$

The expectation value of higher order Gaussian moments can be calculated using the covariance functions, then:

$$S(\omega) = \frac{1}{2\pi}\gamma^2 L^2 \text{sinc}(\frac{\Delta\beta L\omega}{2})\mathcal{F}\{\langle P_r P_p + (P_r Pn + \frac{1}{2}P_r P_p)\exp(-\sigma^2(t-t')^2)\rangle\} \tag{14}$$

$$= \gamma^2 L^2 \text{sinc}(\frac{\Delta\beta L\omega}{2})(P_p P_r \delta(\omega) + \frac{(P_r P_n + P_r P_p/2)}{2\sqrt{\pi}\sigma}\exp(-\frac{\omega}{4\sigma^2})) \tag{15}$$

which is the desired small signal SFG spectrum in Eq. (7).

2.4 Narrowband SFG Regime

If one considers a narrowband SFG scenario and takes as an ansatz that the i-SFG power is negligible, then the expression of c-SFG efficiency can be derived beyond the small signal SFG regime. To do so, rewrite the coupled mode Eqs. (1) (2) as follow:

$$\frac{\partial}{\partial z}A_{SFG}(z,t) = i\gamma A_r(t-\beta z)A_m(z,t) \tag{16}$$

$$(\frac{\partial}{\partial z}+\beta\frac{\partial}{\partial t})(A_m(z,t)A_r^*(t-\beta z)) = i\gamma|A_r(t-\beta z)|^2 A_{SFG}(z,t) \tag{17}$$

Take the ensemble average of both sides, then the time derivative vanishes:

$$\frac{\partial}{\partial z}\langle A_{SFG}(z,t)\rangle = i\gamma\langle A_r(t-\beta z)A_m(z,t)\rangle \tag{18}$$

$$\frac{\partial}{\partial z}\langle(A_m(z,t)A_r(t-\beta z))\rangle = i\gamma\left\langle|A_r(t-\beta z)|^2 A_{SFG}(z,t)\right\rangle \tag{19}$$

We now adopt the approximation that only single-frequency (time-invariant) c-SFG exists:

$$A_{SFG}(z) = A_{SFG}(z,t) = \langle A_{SFG}(z,t)\rangle \tag{20}$$

$$\tag{21}$$

Then:

$$\frac{\partial}{\partial z}A_{SFG}(z) = i\gamma\langle A_r(t-\beta z)A_m(z,t)\rangle \tag{22}$$

$$\frac{\partial}{\partial z}\{\langle A_r(t-\beta z)A_m(z,t)\rangle\} = i\gamma P_r A_{SFG}(z) \tag{23}$$

The solution of this equation is then given by:

$$A_{SFG}(z) = \sin(\gamma\sqrt{P_r}z)\langle A_r(t)A_m(0,t)\rangle/\sqrt{P_r} \tag{24}$$

Since noise light is not correlated with reference light, A_m in the above expression can be replaced by the probe light amplitude A_p (perfection noise rejection is achieved in the narrowband phase-matching limit). If one introduce delay ΔL and group velocity dispersion D_2 to probe light, then:

$$\langle A_r(t)A_p(0,t)\rangle = \sqrt{P_rP_p}\exp\left(-\frac{\beta^2\Delta L^2\sigma^2}{2\sigma^2(1-i\sigma^2D_2)}\right)/(1-i\sigma^2D_2) \tag{25}$$

Then the output SFG amplitude is given by:

$$A_{SFG}(z) = \sqrt{P_p}\sin(\gamma\sqrt{P_r}z)\exp\left(-\frac{\beta^2\Delta L^2\sigma^2}{2\sigma^2(1-i\sigma^2D_2)}\right)/(1-i\sigma^2D_2) \tag{26}$$

This is the desired expression Eq.(10) if the dispersion D_2 is taken to be zero.

2.5 General SFG Regime

An analytical solution to Eq. (1) (2) is given in [10]. However, for chaotic probe and reference light that can only be described statistically, a closed-form analytical solution cannot be obtained. One can instead use the Monte Carlo method to calculate the average conversion efficiencies as follows:

- Numerically generate chaotic waveform for probe and reference light within time interval $[-\beta_{SFG}L, -\beta L]$.
- Use these waveform to calculate the SFG output amplitude $A_{SFG}(L,0)$ [10].
- Repeat the above steps and obtain an array of size K of independent $A_{SFG,k}(L,0), (k=1,2\cdots K)$.
- The total SFG power and c-SFG power are then given by $\frac{1}{K}\sum_k|A_{SFG,k}(L,0)|^2$ and $\frac{1}{K^2}|\sum_k A_{SFG,k}(L,0)|^2$

The simulation code is available upon request.

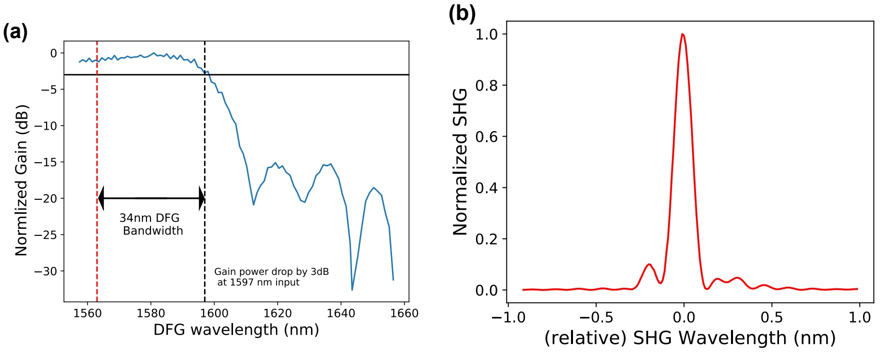

Fig. S2: (a): the normalized DFG gain as a function of the idler wavelength. (b): normalized SHG conversion efficiency as a function of input wavelength.

3 Supplementary Note 3 - Nonlinear Waveguide Information

The y-Cut PPLN nonlinear waveguide for SFG is from HC-photonics, based on proton exchange technology. Details of the waveguide are shown in Fig.S2. The DFG and SFG phase matching spectrum is shown in Fig. S2 with all interacting waves having the same polarization (type-0 phase matching).

Total Insertion Loss	SHG efficiency	Coating Bandwidth(1% reflection)	SHG Temperature Tuning
43%	1000%/W	138nm@780nm, 271nm@1560nm	8K/nm

Wavelength	MFD,-y (um)	MFD,z (um)	NA,-y	NA,z
1558nm	8.82	7.51	0.112	0.085
779nm	5.55	11.68	0.089	0.066

Table S2: Technical specification of the SFG PPLN waveguide. The total insertion loss includes input and output lens coupling (Thorlabs c220tmd-c, c220tmd-b). MFD: mean field diameter. NA: numerical aperture.

4 Supplementary Note 4 - Solid State Delay Scanning for Fast Ranging

It is possible to improve the delay scanning speed by replacing the mechanical delay line with a solid state one, with the schematic shown in Fig.S3(a). It is implemented with two magneto-optical switches (1×4 channel) and two mems (1×8 channels) optical switches. The total scanning range is 4×4×8×1mm =12.8cm, which is only limited by the number of optical switches and the number of channels of each switch. The switching speed of our switch is around 10us for the magneto-optical switch and 0.5ms for the mems switch. The insertion losses of switches are compensated by a fiber amplifier. The ranging result with this new delay line is shown in Fig. S3(b). A narrower bandwidth of probe light is used to increase the probe-reference correlation time as compared to the setup reported in the main text.

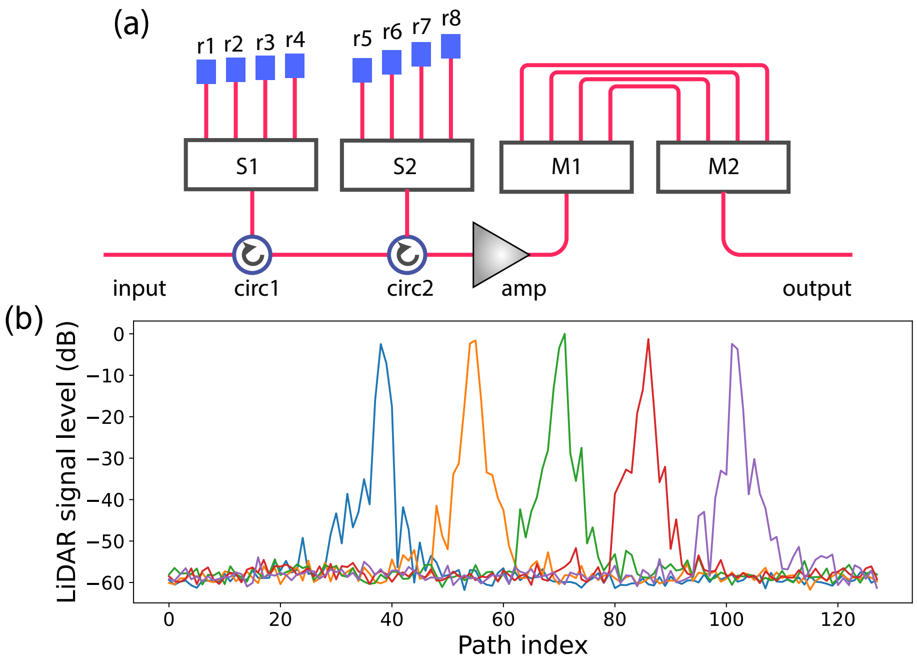

Fig. S3: (a) the schematic of the non-mechanical delay line. circ1,circ2: optical circulators, r1-r8: optical retro-reflectors, s1,s2: solid state optical switches, m1,m2: mems optical switches (only four channels are shown for each switch are shown for simplicity, amp: low power erbium-doped fiber amplifier module. The relative (fiber optical) delay of different channels for s1 and s2 are 1mm and 4mm, respectively. The relative (fiber optical) delay of different channels for the mems switch pair is 1.6cm. (b) the experimental ranging result. Different path indices indicate different combinations of optical switch channels. Adjacent channels have around 1mm (fiber optical) path length difference. The target detection signal is measured in a log scale using balanced homodyne detection and spectrum analyzer.

Supplementary References

[1] Han Liu, Daniel Giovannini, Haoyu He, Duncan England, Benjamin J Sussman, Bhashyam Balaji, and Amr S Helmy. Enhancing lidar performance metrics using continuous-wave photon-pair sources. *Optica*, 6(10):1349–1355, 2019.

[2] Yingwen Zhang, Duncan England, Andrei Nomerotski, Peter Svihra, Steven Ferrante, Paul Hockett, and Benjamin Sussman. Multidimensional quantum-enhanced target detection via spectrotemporal-correlation measurements. *Physical Review A*, 101(5):053808, 2020.

[3] Phillip S Blakey, Han Liu, Georgios Papangelakis, Yutian Zhang, Zacharie M Léger, Meng Lon Iu, and Amr S Helmy. Quantum and non-local effects offer over 40 db noise resilience advantage towards quantum lidar. *Nature communications*, 13(1):1–8, 2022.

[4] Amin Shahverdi, Yong Meng Sua, Ivan Dickson, Malvika Garikapati, and Yu-Ping Huang. Mode selective up-conversion detection for lidar applications. *Optics Express*, 26(12):15914–15923, 2018.

[5] Il-Pyeong Hwang, Seok-jun Yun, and Chang-Hee Lee. Study on the frequency-modulated continuous-wave lidar mutual interference. In *2019 IEEE 19th International Conference on Communication Technology (ICCT)*, pages 1053–1056. IEEE, 2019.

[6] Fan-Yi Lin and Jia-Ming Liu. Chaotic lidar. *IEEE journal of selected topics in quantum electronics*, 10(5):991–997, 2004.

[7] Il-Pyeong Hwang and Chang-Hee Lee. Mutual interferences of a true-random lidar with other lidar signals. *IEEE Access*, 8:124123–124133, 2020.

[8] Liyan Feng, Huazheng Gao, Jianxun Zhang, Minghai Yu, Xianfeng Chen, Weisheng Hu, and Lilin Yi. Fpga-based digital chaotic anti-interference lidar system. *Optics Express*, 29(2):719–728, 2021.

[9] Zheshen Zhang, Sara Mouradian, Franco NC Wong, and Jeffrey H Shapiro. Entanglement-enhanced sensing in a lossy and noisy environment. *Physical review letters*, 114(11):110506, 2015.

[10] Dileep V Reddy, Michael G Raymer, Colin J McKinstrie, Lasse Mejling, and Karsten Rottwitt. Temporal mode selectivity by frequency conversion in second-order nonlinear optical waveguides. *Optics express*, 21(11):13840–13863, 2013.

[11] Benjamin Brecht, Dileep V Reddy, Christine Silberhorn, and Michael G Raymer. Photon temporal modes: a complete framework for quantum information science. *Physical Review X*, 5(4):041017, 2015.
